# NUFIP1 gain of function promotes fibrotic remodeling and reveals a ZNHIT3-linked metabolic vulnerability in idiopathic pulmonary fibrosis models

**DOI:** 10.3389/fimmu.2026.1909302

**Published:** 2026-08-27

**Authors:** Yueyan Lou, Guojun Qian, Zhihan Jiang, Hongyan Zhang, Qing Wei, Lifang Zhao, Xueqing Liu, Shan Xue, Hourong Cai, Handong Jiang

**Affiliations:** 1 Department of Respiratory Medicine, Ren Ji Hospital, Shanghai Jiao Tong University School of Medicine, Shanghai, China; 2 Key Laboratory of Anesthesiology, Shanghai Jiao Tong University, Ministry of Education, Shanghai, China; 3 Department of Anesthesiology, Ren Ji Hospital, Shanghai Jiao Tong University School of Medicine, Shanghai, China; 4 Department of Laboratory Medicine, Ren Ji Hospital, Shanghai Jiao Tong University School of Medicine, Shanghai, China; 5 Department of Respiratory Medicine, The Affiliated Drum Tower Hospital of Nanjing University Medical School, Nanjing, China

**Keywords:** CB-839, fibroblast activation, idiopathic pulmonary fibrosis, NUFIP1, vimentin, ZNHIT3

## Abstract

**Background:**

Fibroblast activation and extracellular matrix deposition are central features of IPF, but the molecular programs that sustain pathogenic fibroblast states remain incompletely defined. NUFIP1 has been implicated in ribonucleoprotein biology and cellular stress responses, yet its role in pulmonary fibrosis is unknown.

**Methods:**

NUFIP1 expression was examined in lung tissues from patients with IPF, bleomycin-induced pulmonary fibrosis in mice, and TGF-β1-stimulated human lung fibroblasts. Gain-of-function studies were performed in MRC-5 fibroblasts and in mice using AAV-mediated NUFIP1 overexpression. The association between NUFIP1 and ZNHIT3 was assessed by co-immunoprecipitation and surface plasmon resonance. The effect of CB-839 on NUFIP1-ZNHIT3 interaction, fibroblast activation, and pulmonary fibrosis was evaluated *in vitro* and *in vivo*. Public bulk and single-cell RNA-seq datasets were analyzed to assess the clinical relevance of NUFIP1 expression in human IPF.

**Results:**

NUFIP1 expression was increased in IPF lung tissue and showed spatial overlap with Vimentin-positive fibrotic regions. AAV-mediated NUFIP1 gain of function was sufficient to induce fibrotic remodeling relative to vector controls, with a magnitude comparable to a separate bleomycin reference group. NUFIP1 associated with ZNHIT3, and CB-839 altered the apparent association under the tested conditions while reducing NUFIP1-associated fibrotic readouts. Public transcriptomic analyses supported a modest donor-level increase in whole-lung NUFIP1 expression and detected NUFIP1 across multiple lineages, including IPF-associated fibroblast states.

**Conclusions:**

These gain-of-function data support a profibrotic effect of increased NUFIP1 in experimental models. ZNHIT3 is a candidate binding partner, and CB-839 reduced NUFIP1-associated fibrotic readouts at the tested dose. Pathway specificity, endogenous necessity, and clinical relevance remain to be established.

## Introduction

Idiopathic pulmonary fibrosis (IPF) is a progressive and frequently fatal interstitial lung disease characterized by irreversible distortion of the lung architecture and declining respiratory function, with a median survival of 3–5 years after diagnosis (1, 2). Although the etiology of IPF remains incompletely understood (3), current antifibrotic agents, including nintedanib and pirfenidone, slow disease progression in some patients but do not reverse established fibrosis or eliminate the need for more precise therapeutic strategies (4–6).

Activated fibroblasts and myofibroblasts are principal effector cells in IPF, producing collagen-rich extracellular matrix and remodeling the injured alveolar interstitium (7, 8). Single-cell studies have revealed disease-enriched fibroblast populations in IPF, but many candidate regulators identified from transcriptomic datasets remain functionally untested. Defining which fibroblast-associated molecules are not merely markers of fibrosis but active contributors to fibroblast activation is essential for developing mechanism-based interventions.

Nuclear FMR1 Interacting Protein 1 (NUFIP1) is a multifunctional RNA-binding and ribonucleoprotein-associated protein involved in nucleocytoplasmic signaling, ribophagy, snoRNP assembly, and transcription-associated DNA damage responses (9–12). NUFIP1 also has emerging links to stromal biology, including cancer-associated fibroblast activation in pancreatic cancer (13), but its role in pulmonary fibrosis has not been defined. NUFIP1 forms a conserved complex with Zinc Finger HIT-Type Containing 3 (ZNHIT3) (14–16), raising the possibility that the NUFIP1-ZNHIT3 may contribute to fibroblast stress adaptation and fibrotic remodeling.

Here, we examined NUFIP1 expression in human IPF tissue, experimental pulmonary fibrosis, and activated lung fibroblasts. We tested the functional consequences of NUFIP1 overexpression *in vitro* and *in vivo*, evaluated the NUFIP1-ZNHIT3 interaction/complex as a candidate binding partner for pulmonary fibrosis, and integrated public human transcriptomic datasets to determine whether NUFIP1 marks a clinically relevant IPF subgroup.

## Materials and methods

### Human samples

The study protocol was approved by the Research Ethics Committee of Renji Hospital, Shanghai Jiao Tong University (KY2024-085B). Patients were diagnosed with IPF according to established clinical practice guidelines and multidisciplinary discussion (17, 18). Individuals with lung cancer, tuberculosis, connective tissue disease, or other non-IPF lung diseases were excluded from the IPF group. Control lung tissues were obtained from histologically normal lung tissue adjacent to surgically resected low-grade malignant pulmonary nodules. Consent procedures were conducted under the approved institutional protocol.

### Animal studies

Male C57BL/6 mice aged 6–8 weeks were maintained under specific pathogen-free conditions at 24 °C with a 12-hour light/dark cycle and 50% ± 10% humidity, with unrestricted access to food and water. For the bleomycin model, mice intratracheally instilled of bleomycin (5 mg/kg) or saline, and lung tissues were collected on day 21 after treatment. Lung-specific overexpression of NUFIP1 was achieved via intratracheally instilled delivery of adeno-associated virus serotype 6 (AAV6) carrying CMV-NUFIP1-3xFLAG-P2A-GFP-WPRE(5x10^11^vg) (OBiO, China), control mice received AAV6-CMV-GFP-WPRE (5x10^11^vg). For pharmacological intervention experiments, AAV-NUFIP1-overexpressing mice were treated with CB-839 at 200 mg/kg orally twice daily for 30 days. The animal experiments were performed in accordance with the Guide for the Care and Use of Laboratory Animals (8th edition, National Institutes of Health, USA) and approved by the Institutional Animal Care and Use Committee (IACUC) of the Shanghai Institute of Materia Medica, China (2024-06-GMY-37).

### Cell culture and treatments

MRC-5 human lung fibroblasts (ATCC, USA) were cultured in Eagle’s Minimum Essential Medium supplemented with 10% fetal bovine serum, Minimum Essential Medium non-essential amino acid solution (Gibco, USA), and sodium pyruvate (Gibco, USA) at 37 °C with 5% CO_2_. Recombinant human transforming growth factor-β1 (TGF-β1; PeproTech, USA) was used to activate fibroblasts at 10 ng/mL for 48 hours, as indicated. CB-839 was applied to cultured fibroblasts at 0.5 μM for 48 hours, as indicated.

### Lentiviral constructs

NUFIP1 overexpression was achieved using lentiviral vectors constructed with pSLenti-EF1-EGFP-P2A-Puro-CMV-MCS-3xFLAG-WPRE (OBiO, China). Negative control vectors were used in parallel experiments.

### Histological analysis

Lung tissues were fixed in 4% paraformaldehyde, dehydrated in ethanol, cleared in xylene, embedded in paraffin, and sectioned at 5 μm. Sections were stained with hematoxylin and eosin (H&E) and Masson’s trichrome for histopathological evaluation.

### Immunohistochemistry

Paraffin-embedded sections were deparaffinized, rehydrated, and subjected to antigen retrieval. Sections were incubated overnight at 4 °C with primary antibodies against NUFIP1 and Vimentin (**Table 1**). After washing, sections were incubated with horseradish peroxidase-conjugated secondary antibodies at 37 °C for 30 minutes.

**Table 1:** Antibodies used for immunohistochemistry.

Target	Source	Company	Catalogue number	Concentration
NUFIP1	Rabbit	Proteintech	12515-1-AP	1:400

### Micro-computed tomography imaging

Mice were anesthetized with 1.5% isoflurane, and CT scans were performed using a Siemens Inveon micro-PET/CT scanner (Siemens Healthineers, Germany) to obtain high-resolution lung images.

### Immunofluorescence staining

Cells and tissue sections were fixed with 4% paraformaldehyde and permeabilized with Triton X-100. Samples were blocked and incubated overnight at 4 °C with primary antibodies (**Table 2**). After washing, samples were incubated with Alexa Fluor 488 or Cyanine5-conjugated secondary antibodies (Thermo Fisher Scientific, USA) for 45 minutes at room temperature. Nuclei were stained with DAPI-containing mounting medium (Beyotime, China). Images were acquired using an Olympus FV3000 confocal microscope (Olympus, Japan).

**Table 2:** Antibodies used for immunofluorescence.

Target	Source	Company	Catalogue number	Concentration
NUFIP1	Rabbit	Proteintech	12515-1-AP	1:250
ZNHIT3	Mouse	Proteintech	68331-1-Ig	1:250
Vimentin	Rabbit	Abcam	Ab92547	1:200

### Western blotting

Cells or lung tissues were lysed in RIPA buffer (Beyotime, China) containing protease inhibitors. Lysates were centrifuged, and protein concentrations were determined using the Pierce BCA Protein Assay Kit (Thermo Fisher Scientific, USA). Proteins (20-40 μg) were separated using sodium dodecyl sulfate-polyacrylamide gel electrophoresis and transferred to polyvinylidene fluoride membranes (Merck Millipore, USA). Membranes were blocked and incubated overnight at 4 °C with primary antibodies (**Table 3**), followed by incubation with secondary antibodies at room temperature for 1 hour. Protein levels were detected using ChemiDoc XRS+ (Bio-Rad, USA).

**Table 3:** Antibodies used for western blotting.

Target	Source	Company	Catalogue number	Concentration
NUFIP1	Rabbit	Proteintech	12515-1-AP	1:750
Vimentin	Rabbit	Abcam	Ab92547	1:1000
β-actin	Rabbit	Beyotime	AF5006	1:1000

### Measurement of hydroxyproline

Levels of the hydroxyproline in the lung tissues were measured using the hydroxyproline assay kit (Nanjing Jiancheng Bioengineering, China), following the manufacturer’s instructions.

### Structural interface analysis

Human NUFIP1 and ZNHIT3 protein sequences were obtained from UniProt (NUFIP1, Q9UHK0; ZNHIT3, Q15649). Structure-guided interface analysis was performed with reference to the NUFIP1-ZNHIT3 structural model and the NMR structure 5L85. Candidate residues and interaction energies derived from structural modeling are summarized in **Table 4**.

**Table 4:** Residues within the modeled interaction region of NUFIP1 and ZNHIT3.

Chain A	Pos A	Set A	Chain B	Pos B	Set B	Energy
1-5L85.A	5	Asp85	2-5L85.B	4	His465	-16.95
1-5L85.A	5	Asp85	2-5L85.B	7	Asn468	-2.60
1-5L85.A	38	Asp118	2-5L85.B	14	Arg475	-24.20
1-5L85.A	47	Met127	2-5L85.B	6	Arg467	-0.70
1-5L85.A	63	Cys143	2-5L85.B	12	Cys473	-0.70
1-5L85.A	68	Glu148	2-5L85.B	4	His465	-18.39

Structural analysis of the NUFIP1-ZNHIT3 complex was based on the NMR structure 5L85. Set A corresponds to residues from ZNHIT3, and Set B represents residues from NUFIP1. Interaction energy is shown in kcal/mol; more negative values indicate stronger predicted binding.

### Measurement of glutamine and glutamate levels

We used a glutamine assay kit (Solarbio, China) and a glutamate assay kit (Solarbio, China) to determine the concentrations of the respective substances within the cells. In brief, after cell lysis, centrifugation was performed to collect the supernatant. Subsequently, gradient dilution was carried out using standard solutions. Then, the absorbance of the gradient-diluted standard solutions and samples was measured using a Synergy H1 microplate reader (BioTek, USA). A standard curve was constructed based on the absorbance values of the gradient-diluted standard solutions. The level of the corresponding substance in the samples was predicted using the standard curve.

### Surface plasmon resonance

Surface plasmon resonance (SPR) assays were performed using a Biacore 1K system (Cytiva, USA). NUFIP1 was diluted in 50 μg/mL sodium acetate buffer (pH 5.0) and immobilized on a CM5 sensor chip by covalent coupling. CB-839 and ZNHIT3 were passed over immobilized NUFIP1 at a flow rate of 30 μl/min. For kinetic analyses of ZNHIT3-NUFIP1 binding, ZNHIT3 was dissolved in running buffer at concentrations ranging from 0.03125 to 1 μM. Association and dissociation phases were each monitored for 60 seconds. Kinetic parameters were calculated using the steady-state affinity fit model according to the manufacturer’s software manual.

### Co-immunoprecipitation

To investigate the association between NUFIP1 and ZNHIT3, MRC-5 cells ectopically expressing NUFIP1 were treated with CB-839 (0.5 μM) for 24 h and lysed in RIPA buffer (Beyotime, P0013D) with the addition of protease inhibitor. For analyzing the association between ZNHIT3 and NUFIP1, the cell lysates were incubated with a NUFIP1 antibody (1 μL/sample) overnight, followed by incubation with 10 μL protein A/G beads (Santa Cruz, SC-2003) for another 3 h at 4 °C. The protein-containing beads were washed three times with PBS and subjected to Western Blot by using specific antibodies for NUFIP1 (Proteintech, 12515-1-AP, 1:750), ZNHIT3 (Proteintech, 68331-1-Ig, 1:2000).

### Public bulk and single-cell transcriptomic analysis

Public datasets GSE213001 (19) and GSE135893 (20) were obtained from the Gene Expression Omnibus. GSE213001 contains 139 regional lung samples (62 IPF, 41 non-diseased controls, 26 non-IPF ILD, and 10 chronic lung allograft dysfunction samples). The present analysis included only IPF (20 donors) and non-diseased control samples (14 donors); non-IPF ILD and allograft samples were excluded. Normalized logRPKM values were averaged across available regional samples within each donor. Donor-level NUFIP1 expression was compared using a two-sided Wilcoxon rank-sum test. Raw counts were additionally summed within donor and analyzed with DESeq2 (v1.46.0) as a sensitivity analysis. Clinical variables were obtained from the GEO series matrix; values recorded as proportions were converted to percentages. Baseline FVC was available for 19 IPF donors and DLCO for 16; survival and longitudinal FVC decline were not available.

**Figure 1:**
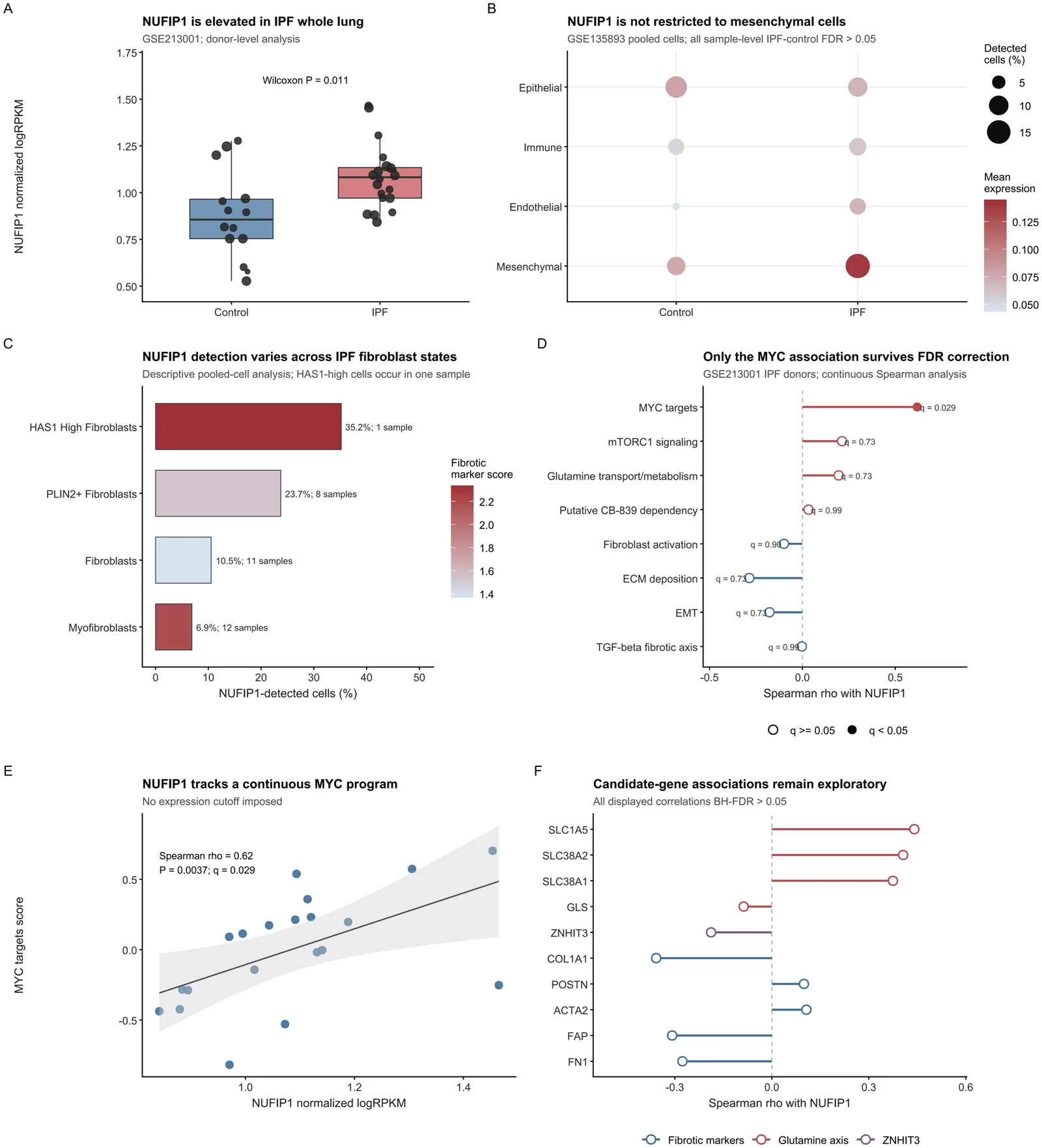
Public transcriptomic analyses support NUFIP1 upregulation and a continuous MYC-linked program in IPF. **(A)** Donor-level NUFIP1 normalized logRPKM in GSE213001 non-diseased controls (n = 14 donors; 41 regional samples) and IPF (n = 20 donors; 62 regional samples). Point size indicates the number of regional samples; the P value is from a two-sided Wilcoxon rank-sum test. **(B)** NUFIP1 across major GSE135893 lineages in control and IPF. Dot size represents the pooled percentage of NUFIP1-detected cells and color represents pooled mean log1p(CP10K); sample-level IPF-control comparisons were tested by two-sided Wilcoxon tests with BH correction and all FDR values were greater than 0.05. **(C)** Descriptive pooled-cell NUFIP1 detection in IPF fibroblast-lineage states. Labels give detection frequency and contributing sample count; color denotes the mean fibroblast activation score. **(D)** Continuous Spearman correlations between donor NUFIP1 and eight prespecified signature scores in GSE213001 IPF. Filled symbols indicate BH-FDR < 0.05. **(E)** Continuous association between NUFIP1 and the Hallmark MYC targets V1 score; the line and shaded band show an ordinary least-squares fit and 95% confidence interval for visualization, whereas inference used Spearman correlation. **(F)** Spearman correlations between NUFIP1 and selected glutamine, ZNHIT3, and fibrotic-marker genes. BH correction was applied across all 27 tested genes; none was significant.

For GSE213001 IPF donors, signature scores were calculated as the mean of gene-wise z scores for genes present in each frozen gene set. Continuous associations with NUFIP1 were tested using two-sided Spearman correlations. Benjamini-Hochberg correction was applied across the eight prespecified **Figure 1** pathways and separately across all 27 tested marker genes. A parsimonious linear sensitivity model adjusted for age and sex using HC3 robust standard errors. An exploratory extended model additionally included smoking status and ordinal disease severity using HC1 standard errors; its results were interpreted cautiously because only 20 IPF donors were available. Gaussian mixture models with one to three components and equal or variable variance were compared by Bayesian information criterion using mclust (v6.1.1), with leave-one-out stability analysis. Pathway scores were also clustered by Ward’s method and the number of clusters selected by mean silhouette width. A fixed random seed of 20260728 was used.

Analyses were performed in R 4.4.2 using GEOquery 2.74.0, DESeq2 1.46.0, AnnotationDbi 1.68.0, org.Hs.eg.db 3.20.0, readr 2.1.5, ggplot2 4.0.3, mclust 6.1.1, sandwich 3.1-1, lmtest 0.9-40, cluster 2.1.6, and patchwork 1.3.0, and in Python 3.12.13 using standard-library streaming scripts. Exact code, package versions, frozen signature gene sets, data checksums, sample characteristics, intermediate tables, and run instructions are available at **Supplementary Source Data 2**.

### Statistical analysis

Data are presented as mean ± standard deviation. An unpaired, two-sided Student’s t-test was used to compare two independent groups after assessment of normality and homogeneity of variances. One-way analysis of variance followed by *post hoc* testing was used for comparisons among multiple groups. Nonparametric public transcriptomic analyses were performed using Wilcoxon rank-sum tests and Spearman correlation, as appropriate. A *P* value < 0.05 was considered statistically significant. Statistical analyses were performed using GraphPad Prism 10 (GraphPad Software, USA), R, and Python.

## Results

### NUFIP1 is increased in fibrotic regions of human IPF lung

**Figure 2:**
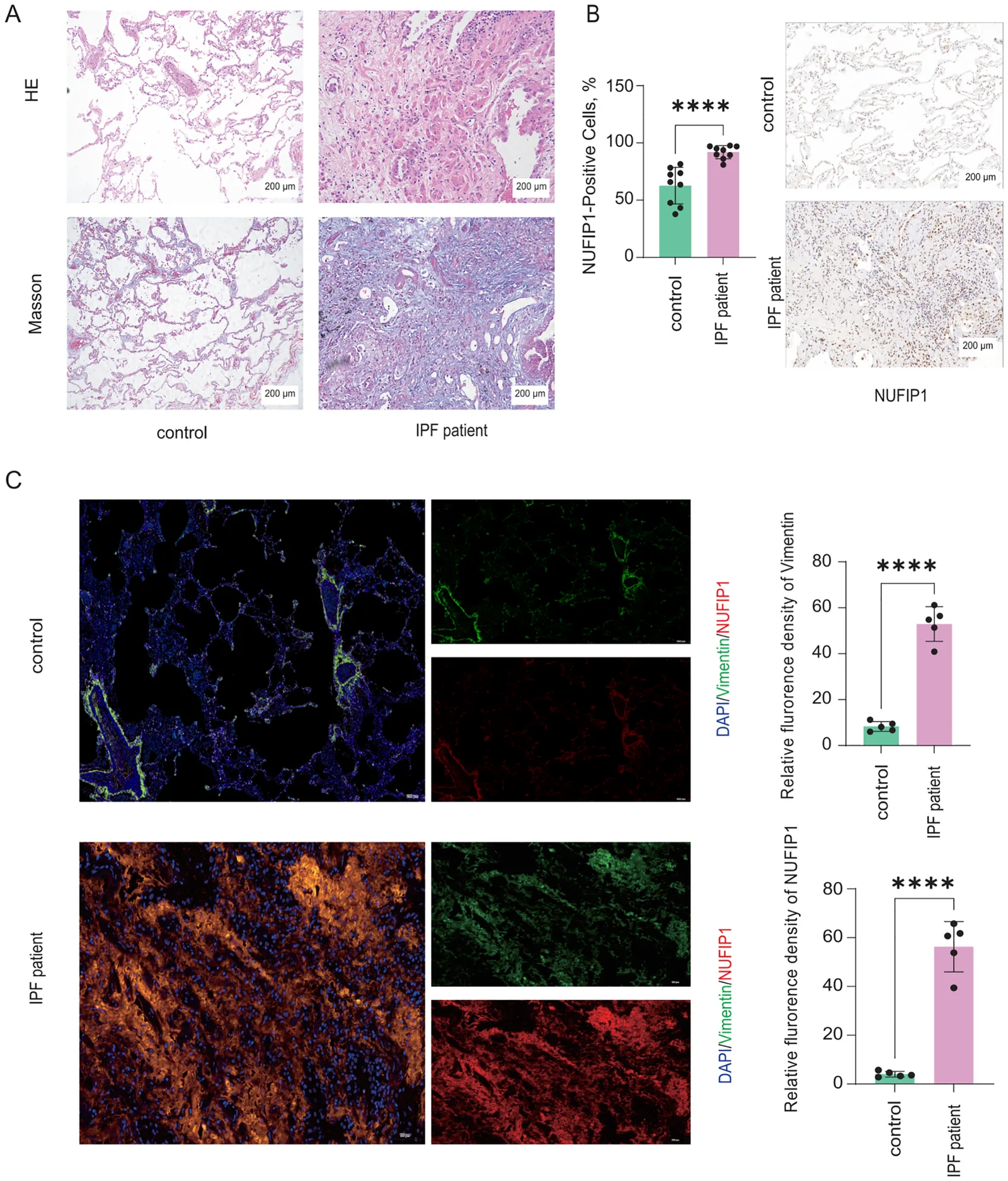
Elevated NUFIP1 expression in lung tissues from patients with IPF. **(A)** Representative H&E and Masson’s trichrome staining of lung tissues from control individuals and patients with IPF. scale bars, 200 μm. **(B)** Immunohistochemistry staining showing NUFIP1 expression in lung tissues from control individuals and patients with IPF. scale bars, 200 μm. **(C)** Immunofluorescence staining of lung sections from control individuals and patients with IPF, showing nuclei (DAPI, blue), Vimentin (green), and NUFIP1 (red). scale bars, 100 μm. Values are presented as mean ± standard deviation with significant levels denoted as 'ns' indicating not significant, *P < 0.05, **P < 0.01, ***P < 0.001, ****P < 0.0001.

We first verified the pathological alterations in lung tissues from control subjects and IPF patients using H&E and Masson’s trichrome staining (**Figure 2A**). Subsequently, immunohistochemical staining analysis revealed that NUFIP1 expression was significantly upregulated in lung tissues of patients with IPF relative to control subjects (**Figure 2B**). Immunofluorescence analysis further demonstrated co-staining of NUFIP1 and Vimentin in fibrotic regions (**Figure 2C**). These findings indicate that NUFIP1 is enriched within the fibrotic microenvironment of human IPF lung.

### NUFIP1 expression is induced across experimental models of fibroblast activation and pulmonary fibrosis

**Figure 3:**
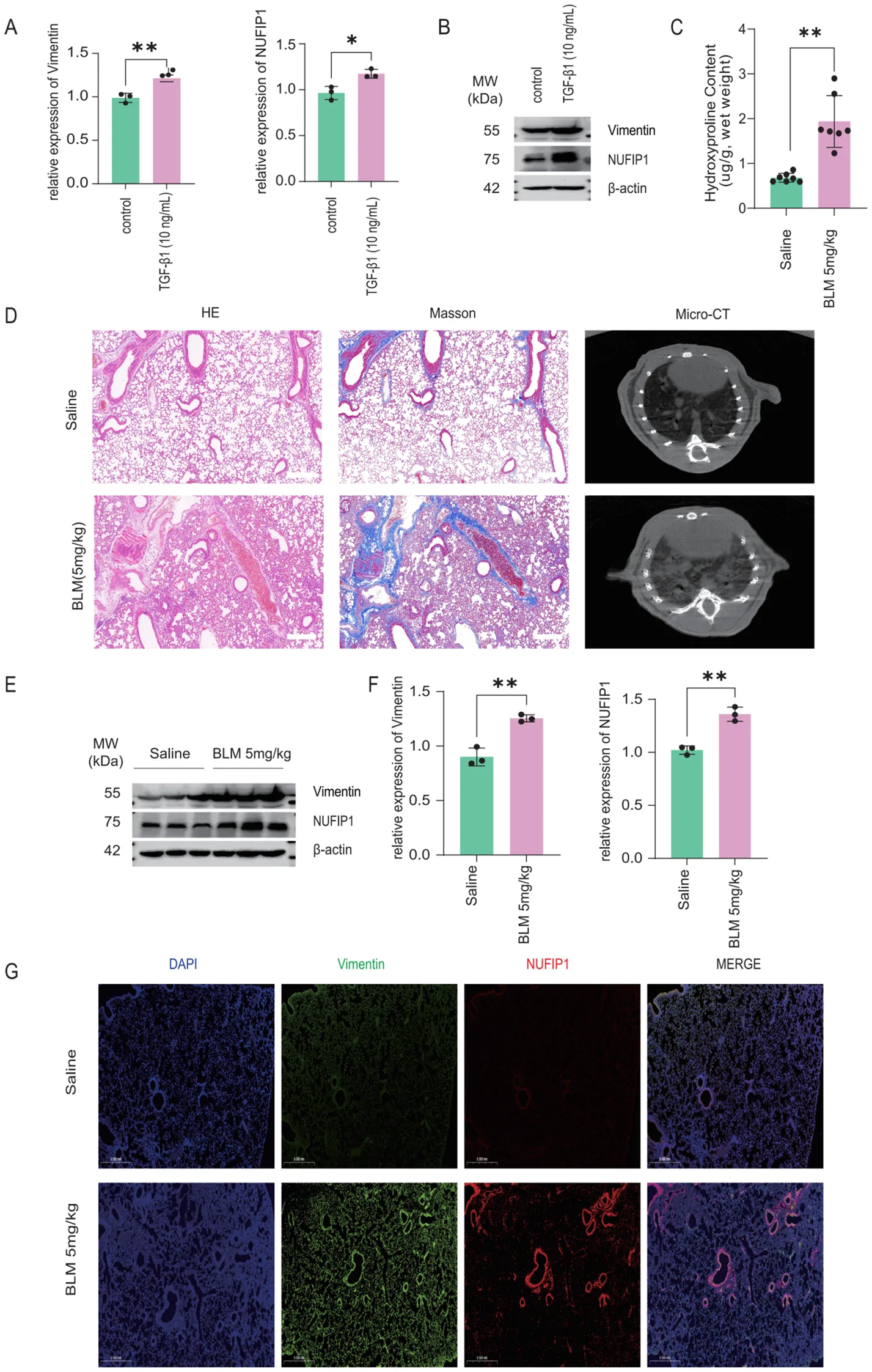
NUFIP1 is upregulated in activated lung fibroblasts and bleomycin-induced pulmonary fibrosis. **(A, B)** MRC-5 fibroblasts were treated with TGF-β1 (10 ng/mL) for 48 hours, Vimentin and NUFIP1protein levels were assessed by western blotting. C57BL/6 mice were intratracheally administered bleomycin (5 mg/kg) or saline, n = 8-10. Lung tissues were collected on day 21 after treatment. **(C)** The hydroxyproline content in lung tissue among groups. **(D)** Representative H&E and Masson’s trichrome staining of lung sections, scale bars, 200 μm; together with representative micro-CT images of mouse lungs from the indicated groups. **(E, F)** Western blot analysis of NUFIP1 and Vimentin protein levels in lung tissues. **(G)** Immunofluorescence staining of lung sections from saline- and bleomycin-treated mice at day 21, showing nuclei (DAPI, blue), Vimentin (green), and NUFIP1 (red). scale bars, 0.5 mm. Values are presented as mean ± standard deviation with significant levels denoted as 'ns' indicating not significant, *P < 0.05, **P < 0.01, ***P < 0.001, ****P < 0.0001.

To determine whether NUFIP1 upregulation was conserved in experimental fibrotic settings, we examined TGF-β1-stimulated MRC-5 human lung fibroblasts and bleomycin-induced pulmonary fibrosis in mice. TGF-β1 treatment increased NUFIP1 expression in parallel with Vimentin induction in MRC-5 cells (**Figures 3A, B**). Consistently, mice subjected to bleomycin-induced pulmonary fibrosis (**Figures 3C, D**), showed elevated NUFIP1 and Vimentin expression compared with saline-treated controls (**Figures 3E–G**). Together, these results show that NUFIP1 upregulation accompanies fibroblast activation and pulmonary fibrosis across complementary models.

### NUFIP1 gain of function was sufficient to induce fibrotic remodeling versus vector and produced a BLM-comparable phenotype

**Figure 4:**
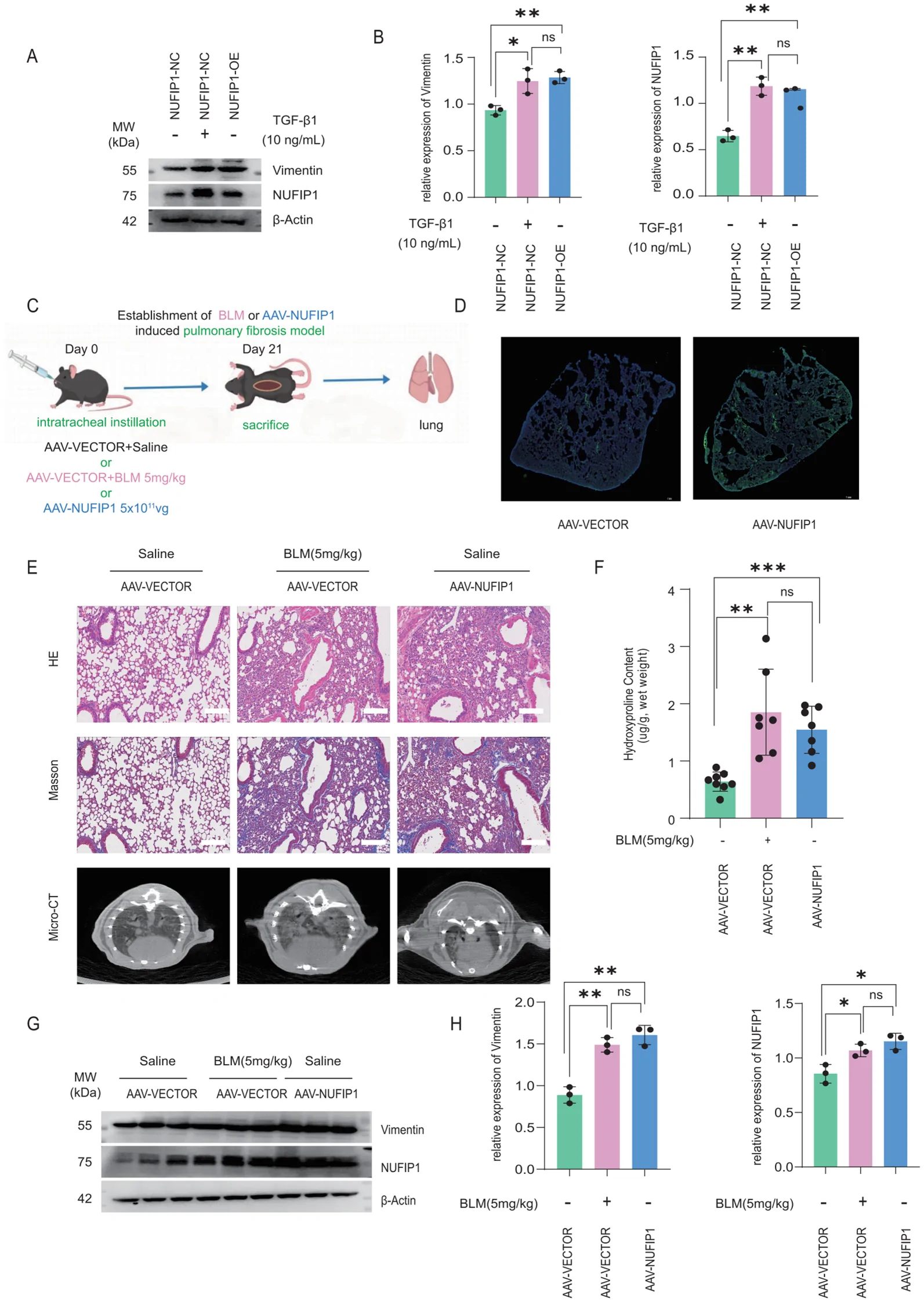
NUFIP1 overexpression promotes lung fibroblast activation and pulmonary fibrosis. **(A, B)** MRC-5 fibroblasts with NUFIP1 overexpression (NUFIP1-OE) or negative control (NC) were treated with TGF-β1 (10 ng/mL). After 48 hours, Vimentin and NUFIP1 protein levels were measured by western blotting. **(C)** Schematic illustration of AAV-NUFIP1 overexpression and bleomycin (BLM)-induced pulmonary fibrosis mouse models. Mice were divided into three groups: AAV-Vector plus saline, AAV-Vector plus BLM, and AAV-NUFIP1. Animals received intratracheal instillation of 5 × 10¹¹ vg AAV-Vector or AAV-NUFIP1; n = 8-10. Within the AAV-Vector cohorts, mice were further treated with intratracheal saline or 5 mg/kg BLM. Mice were sacrificed 21 days after. **(D)** Lung tissues were harvested on day 3 after transfection from mice receiving green fluorescent AAV-Vector and AAV-NUFIP1 for immunofluorescence staining. Nuclei were stained with DAPI (blue). scale bars, 1 mm. **(E)** Representative H&E and Masson’s trichrome staining of lung sections, scale bars, 200 μm; together with representative micro-CT images of mouse lungs from the indicated groups. **(F)** The hydroxyproline content in lung tissue among groups. **(G, H)** Western blot analysis of Vimentin and NUFIP1 protein levels in lung tissues from the indicated groups. Values are presented as mean ± standard deviation with significant levels denoted as 'ns' indicating not significant, *P < 0.05, **P < 0.01, ***P < 0.001, ****P < 0.0001.

We next tested whether NUFIP1 could functionally promote fibroblast activation. Lentiviral NUFIP1 overexpression in MRC-5 fibroblasts increased Vimentin production, in the absence of exogenous TGF-β1 stimulation (**Figures 4A, B**). These data suggest that NUFIP1 can promote a fibroblast activation program.

We subsequently evaluated the profibrotic effect of NUFIP1 *in vivo* by overexpressing the protein in C57BL/6 mice using adeno-associated virus (AAV) (**Figure 4C**).We administered AAV-NUFIP1 to the mouse lungs, and lung tissues were harvested 3 days post-transfection for immunofluorescence staining to verify successful pulmonary transduction of AAV-NUFIP1(**Figure 4D**). NUFIP1-overexpressing was capable of triggering the development of pulmonary fibrosis like mice receiving bleomycin, as shown by H&E and Masson’s trichrome staining, and furthermore, micro-CT further confirmed the formation of pulmonary fibrosis (**Figure 4E**). Consistently, quantitative analysis of hydroxyproline content in lung tissues from the three groups demonstrated that overexpression of NUFIP1 induces pulmonary fibrosis in mice (**Figure 4F**). NUFIP1 overexpression also increased Vimentin expression in lung tissue (**Figures 4G, H**). Compared with AAV-vector controls, AAV-mediated NUFIP1 gain of function was sufficient to induce histological remodeling and increase lung hydroxyproline. The magnitude of these readouts was comparable to that in the separate bleomycin reference group. These gain-of-function results support a functional contribution of NUFIP1 to fibrotic remodeling.

### NUFIP1 associates with ZNHIT3 and is pharmacologically attenuated by CB-839

**Figure 5:**
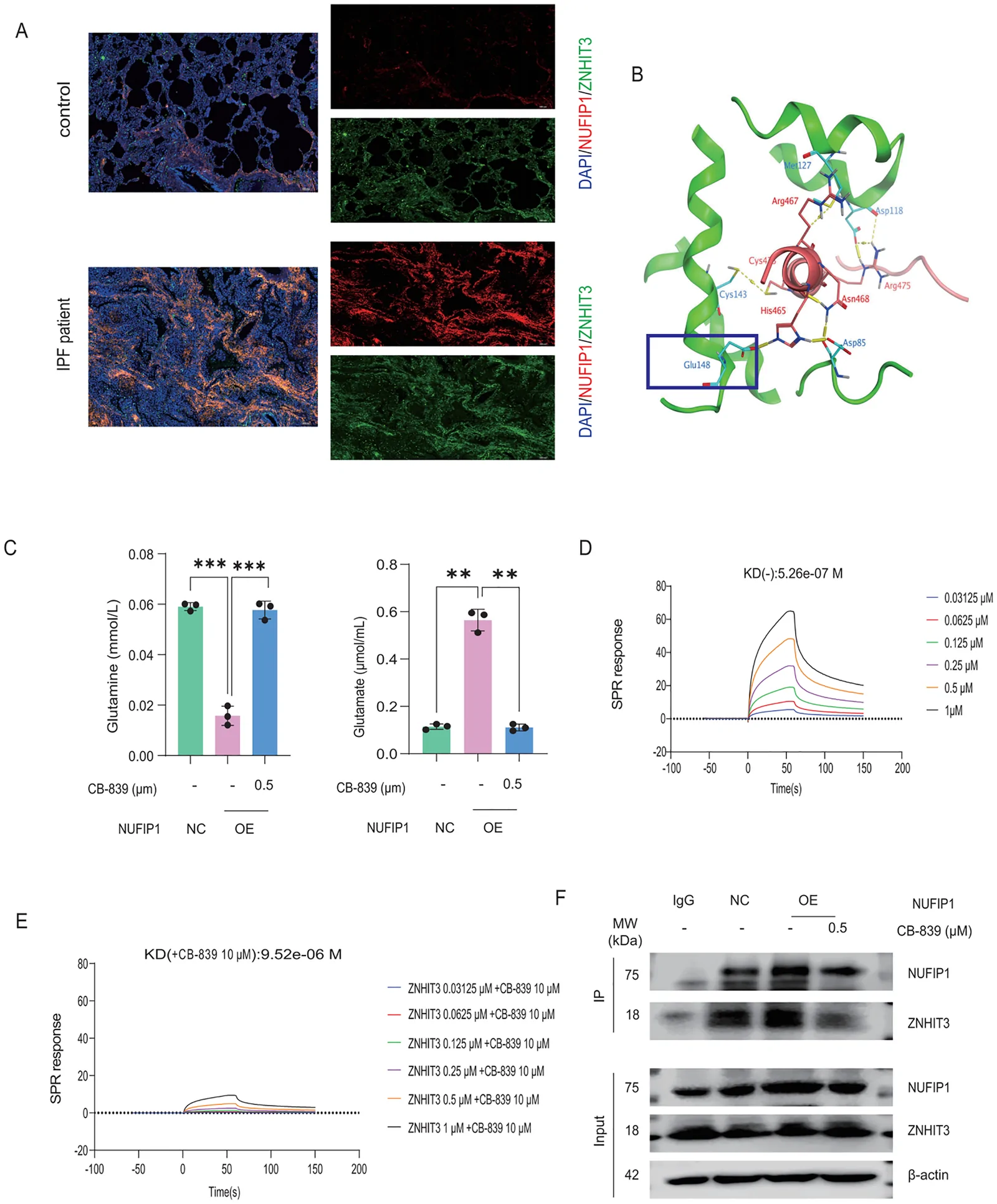
NUFIP1 associates with ZNHIT3, with an altered apparent association under CB-839 treatment. **(A)** Immunofluorescence staining of lung tissues from control and IPF patients. ZNHIT3 (green), NUFIP1 (red), and nuclei (DAPI, blue) are shown. scale bars, 200 μm. **(B)** Using the UniProt database (accession numbers: Q9UHK0 for NUFIP1, Q15649 for ZNHIT3), we identified and analyzed the human protein sequences. Structural alignment revealed that the docked NUFIP1-ZNHIT3 complex closely corresponds to the NMR structure 5L85, with the Cterminal helix of NUFIP1 (red) adopting a binding conformation that aligns well with the C-terminal double helix of ZNHIT3 (green). **(C)** Glutamine and glutamate levels in MRC-5 fibroblasts overexpressing NUFIP1 or controls. **(D, E)** The kinetic binding constant for the interaction between ZNHIT3 and NUFIP1 was determined using surface plasmon resonance (SPR), both in the presence and absence of CB-839. **(F)** Co-IP analysis of ZNHIT3 association with NUFIP1 in MRC-5 cells overexpressing NUFIP1 or controls, respectively, and treated with CB-839 (0.5 μM) for 24 hours. The values are presented as the means ± standard deviations.

NUFIP1 and ZNHIT3 form a conserved structural complex involved in ribonucleoprotein biology (11). Immunofluorescence analysis of lung tissues from IPF patients and control subjects revealed that ZNHIT3 expression was detectable (**Figure 5A**). Accordingly, the mode of interaction between NUFIP1 and ZNHIT3, as well as whether their interplay is associated with pulmonary fibrogenesis, aroused our interest. Structure-guided interface analysis based on UniProt sequences and the 5L85 structural model identified acidic residues, including aspartate and glutamate, at the modeled interaction region of NUFIP1 and ZNHIT3 (**Figure 5B**; **Table 4**).

CB-839, a glutaminase inhibitor that limits glutamine-to-glutamate conversion, as a pharmacological probe of this metabolic axis. We first quantified glutamine and glutamate contents in NUFIP1-overexpressing MRC-5 cells. The results demonstrated that CB-839 blocks the conversion of glutamine to glutamate and decreases glutamate levels in NUFIP1-overexpressing cells (**Figure 5C**). SPR assays showed that CB-839 reduced the apparent affinity between immobilized NUFIP1 and ZNHIT3 under the tested conditions, with the measured KD shifting from 5.26 × 10^−7^ M to 9.52 × 10^−6^ M in the presence of CB-839 (**Figures 5D, E**; **Table 5**). We further performed Co-IP assays and confirmed that NUFIP1 overexpression promotes its binding to ZNHIT3, whereas CB-839 attenuates the apparent association between NUFIP1 and ZNHIT3 (**Figure 5F**). NUFIP1 gain of function was associated with lower steady-state intracellular glutamine and higher glutamate, whereas CB-839 shifted both measurements in the opposite direction under the tested conditions. ZNHIT3 was detected in NUFIP1 immunoprecipitates; however, because recovered IP-NUFIP1 differed among conditions, the CB-839-associated change is described as a reduced apparent association rather than definitive disruption of the complex.

**Table 5:** SPR-derived binding parameters for the NUFIP1-ZNHIT3 interaction.

Ligand	Analyte	Ka (1/Ms)	Kd (1/s)	KD (M)
NUFIP1	ZNHIT3	1.66e+04	8.74e-03	5.26e-07
NUFIP1	ZNHIT3 + CB-839 10 μM	7.28e+02	6.93e-03	9.52e-06

KD, dissociation constant; Ka, association rate constant; Kd, dissociation rate constant.

### CB-839 reduces NUFIP1-associated fibroblast activation and fibrotic remodeling

**Figure 6:**
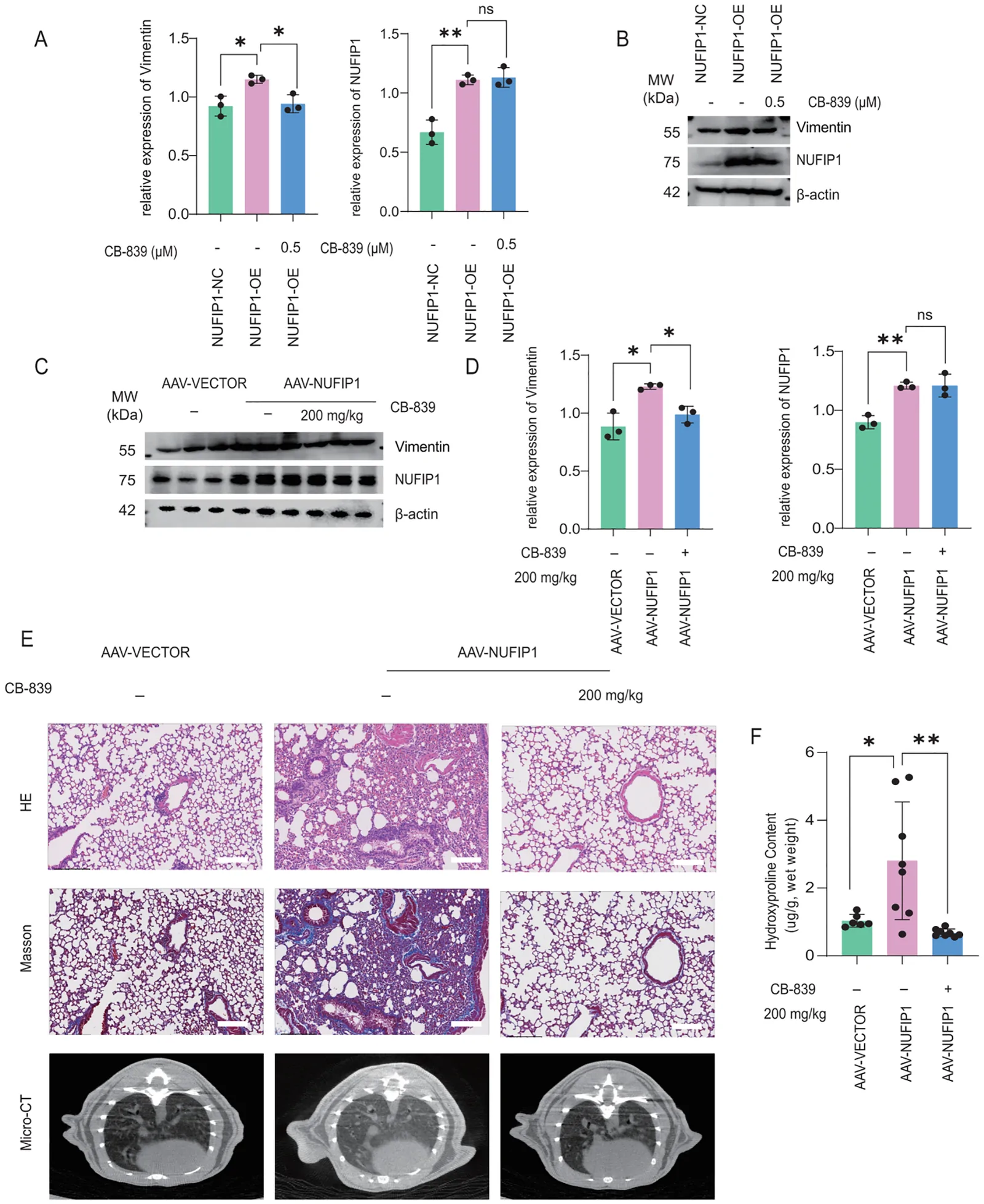
CB-839 attenuates NUFIP1-associated fibroblast activation and pulmonary fibrosis. **(A, B)** MRC-5 fibroblasts overexpressing NUFIP1 (NUFIP1-OE) or negative control (NC) were treated with CB-839 (0.5 μM). Western blot analysis of Vimentin and NUFIP1 protein levels was performed after 48 hours. **(C, D)** Western blot analysis of Vimentin and NUFIP1 protein levels in lung tissues from AAV-VECTOR mice, AAV-NUFIP1 mice, and AAV-NUFIP1 mice treated with CB-839 (200 mg/kg orally twice daily for 30 days); n = 8-10. **(E)** Representative H&E and Masson’s trichrome staining of lung sections, scale bars, 200 μm; and representative micro-CT images of mouse lungs from the indicated groups. **(F)** The hydroxyproline content in lung tissue among groups. Values are presented as mean ± standard deviation.

We next investigated whether pharmacological attenuation by CB-839 could ameliorate NUFIP1-related fibrotic phenotypes. Consistent with this notion, CB-839 treatment reduced Vimentin expression in NUFIP1-overexpressing fibroblasts (**Figures 6A, B**).

To evaluate the pharmacological intervention at the tested dose *in vivo*, CB-839 was administered to mice developing AAV-NUFIP1-induced pulmonary fibrosis. Treatment with CB-839 (200 mg/kg) alleviated fibrotic pathology and lowered Vimentin expression relative to vehicle-treated AAV-NUFIP1 control mice (**Figures 6C–F**). Safety assessments revealed no obvious adverse impacts on body weight, hepatic and renal function, coagulation profiles, or cardiac injury markers (**Supplementary Figures 1A, B**). Collectively, at the tested dose, CB-839 reduced Vimentin abundance in NUFIP1 gain-of-function fibroblasts and decreased selected histological and biochemical fibrotic readouts in AAV-NUFIP1 mice.

### Public transcriptomic analyses support NUFIP1 upregulation but not a validated NUFIP1-defined clinical subtype

Donor-level analysis of GSE213001 showed higher normalized NUFIP1 expression in IPF lungs (20 donors; 62 regional samples) than in non-diseased control lungs (14 donors; 41 regional samples; two-sided Wilcoxon P = 0.011; **Figure 1A**). A donor-pseudobulk DESeq2 sensitivity analysis showed the same direction but did not reach statistical significance (log2 fold change = 0.085, FDR = 0.330), indicating that the external bulk evidence is supportive but modest. In GSE135893, NUFIP1 was detected across epithelial, immune, endothelial, and mesenchymal lineages rather than being fibroblast-specific. Mesenchymal cells had the highest pooled NUFIP1 expression and detection frequency in IPF, but no sample-level IPF-control lineage comparison remained significant after multiple-testing correction (all FDR > 0.05; **Figure 1B**). Within IPF fibroblast-lineage cells, NUFIP1 detection was highest in HAS1-high fibroblasts (35.2%; 179 cells from one sample) and PLIN2+ fibroblasts (23.7%; 1,167 cells from eight samples), although these pooled-cell summaries are descriptive (**Figure 1C**).

Among the 20 GSE213001 IPF donors, continuous NUFIP1 expression correlated with the Hallmark MYC targets V1 score (Spearman rho = 0.618, P = 0.0037, BH-FDR = 0.029), whereas mTORC1, glutamine transport/metabolism, putative CB-839 dependency, fibroblast activation, ECM deposition, EMT, and TGF-beta fibrotic signatures were not significant after correction (**Figures 1D, E**).

The MYC association retained a positive direction after adjustment for age and sex (beta per SD NUFIP1 = 0.245, HC3 P = 0.070) and in an exploratory model additionally including smoking and disease severity (beta = 0.288, HC1 P = 0.011, pathway-level FDR = 0.091; **Supplementary Figure 2C**). Individual glutamine and fibrotic-marker correlations were also non-significant after BH correction (all FDR >= 0.546; **Figure 1F**).

A data-driven Gaussian mixture model favored two NUFIP1 components, but the higher component contained only 2 of 20 donors; 18 of 20 leave-one-out fits selected two components (**Supplementary Figures 2A, B**). Unsupervised pathway clustering was not associated with NUFIP1 (Kruskal-Wallis P = 0.706). NUFIP1 was not associated with cross-sectional disease severity, baseline FVC, or baseline DLCO (all FDR > 0.85; **Supplementary Figure 2D**), and survival and longitudinal FVC decline were not available. Thus, the public data support disease-associated NUFIP1 upregulation and a continuous MYC-linked molecular program, but do not establish a discrete prognostic subgroup or a transcriptomic predictor of CB-839 response.

## Discussion

This study identifies NUFIP1 as a previously unrecognized regulator of fibroblast activation in pulmonary fibrosis. NUFIP1 was increased in fibrotic regions of human IPF lung, induced in experimental models of fibroblast activation, and sufficient to enhance Vimentin production in lung fibroblasts. *In vivo*, AAV-mediated NUFIP1 overexpression is sufficient to induce pulmonary fibrotic remodeling, supporting that NUFIP1 actively promotes fibrogenesis rather than merely correlating with fibrotic lesions.

MRC-5 is a fetal lung fibroblast line and therefore does not reproduce the age-associated epigenetic, senescence, and metabolic features of primary adult or IPF-derived fibroblasts. The *in vitro* findings should be interpreted as mechanistic proof-of-concept and require validation in primary adult normal and IPF lung fibroblasts. Mechanistically, our data implicate the NUFIP1-ZNHIT3 complex as a candidate effector axis. NUFIP1 associated with ZNHIT3 in fibroblasts, and CB-839 appears to weaken the physical association together with Vimentin expression and histological fibrosis. Because CB-839 primarily inhibits glutaminase (21, 22), these findings should be interpreted as evidence that a glutamine-glutamate-linked pharmacological intervention can suppress NUFIP1-associated fibrotic phenotypes. Direct proof that free glutamate stabilizes the NUFIP1-ZNHIT3 complex will require intracellular glutamate measurements, glutamate or α-ketoglutarate rescue, GLS genetic perturbation, and mutational validation of the predicted interface (23–26).

The public transcriptomic analyses refine the interpretation of our experimental findings. NUFIP1 was increased at the donor level in an independent whole-lung cohort and was detectable in selected IPF-associated fibroblast states; however, it was not fibroblast-specific, and continuous variation in NUFIP1 across IPF lungs was not accompanied by higher ECM, fibroblast activation, EMT, or TGF-beta signature scores. This does not demonstrate that the experimental effect is absent in patients. The overexpression experiments test whether increased NUFIP1 is sufficient to activate fibroblasts under controlled conditions, whereas cross-sectional whole-lung data test covariation across heterogeneous, regionally sampled, late-stage tissues. Bulk expression is influenced by cell composition, fibrotic programs may already be saturated across advanced IPF explants, and NUFIP1 activity may depend on protein abundance, localization, glutamate availability, or NUFIP1-ZNHIT3 complex formation rather than NUFIP1 mRNA alone. The public datasets do not measure these quantities. Accordingly, the lack of a bulk ECM correlation should be interpreted as a limitation on patient-level extrapolation, not as independent confirmation of the proposed mechanism (13, 27). The only pathway association that survived multiple-testing correction was a continuous relationship between NUFIP1 and MYC targets. A data-driven model also identified a two-donor high-expression tail, but the small cohort and absence of replication preclude defining a clinically meaningful NUFIP1-high subtype. Moreover, NUFIP1 was unrelated to available baseline FVC, DLCO, or cross-sectional severity, and the datasets lacked survival, longitudinal decline, glutamate, protein-interaction, and CB-839 response measurements.

Therefore, these analyses support NUFIP1 disease relevance and generate a MYC-linked metabolic hypothesis, but they do not establish NUFIP1 as a prognostic biomarker or predict sensitivity to glutaminase inhibition. Prospective cohorts with longitudinal outcomes and pharmacodynamic measurements will be required to test those possibilities.

NUFIP1 was initially characterized as a nuclear protein that interacts with Fragile X Mental Retardation Protein and shuttles between the nucleus and cytoplasm (9). Subsequent studies have linked NUFIP1 to ribophagy, C/D box snoRNP assembly, and transcription-associated DNA damage repair (10–12). More recently, NUFIP1-dependent autophagy was shown to support the metabolic activity of cancer-associated fibroblasts in pancreatic cancer (13), consistent with the concept that NUFIP1 can shape stromal cell function under stress. NUFIP1-related interventions have also been reported to modulate apoptosis-related injury in non-pulmonary experimental settings (28). Our findings extend this biology to lung fibrosis and identify fibroblast activation as a disease-relevant output of NUFIP1 activity.

NUFIP1 forms a heterodimer with ZNHIT3, and studies in yeast and human cells have shown that this interaction contributes to protein stability, snoRNA homeostasis, and normal growth (14, 15). Structural studies further indicate that the NUFIP1-ZNHIT3 interaction is conserved and selective (16, 29). In the present study, structural modeling identified acidic residues within a modeled interaction region, while SPR and co-immunoprecipitation assays showed that CB-839 reduced the apparent NUFIP1-ZNHIT3 association. These data support the NUFIP1-ZNHIT3 complex as a candidate node linking fibroblast activation to metabolic regulation, but they do not yet prove that glutamate directly bridges or stabilizes the complex.

Several limitations should be acknowledged. First, the study relies primarily on gain-of-function approaches because complete NUFIP1 ablation was not tolerated in preliminary *in vivo* and *in vitro* attempts. Partial knockdown, inducible depletion, or CRISPR interference strategies will be needed to establish loss-of-function causality while avoiding developmental or homeostatic lethality. Second, CB-839 is a glutaminase inhibitor and may affect multiple glutamine-dependent pathways beyond NUFIP1-ZNHIT3 complex formation. Genetic GLS perturbation and metabolite rescue experiments will be important to define on-target and pathway-specific effects. Third, public transcriptomic datasets do not contain glutamate measurements, NUFIP1-ZNHIT3 protein-interaction readouts, or CB-839 treatment response. These analyses should therefore be interpreted as patient-level validation and hypothesis-generating stratification rather than direct mechanistic proof.

## Conclusions

NUFIP1 is increased in human IPF tissue, and NUFIP1 gain of function is sufficient to enhance selected fibrotic phenotypes in experimental models. ZNHIT3 is a candidate NUFIP1-binding partner, while CB-839 suppresses NUFIP1-associated fibrotic readouts at the tested dose. Loss-of-function studies, ZNHIT3/GLS epistasis, metabolic-flux and rescue experiments, and longitudinal clinical cohorts are required before establishing endogenous necessity, pathway specificity, prognostic relevance, or therapeutic utility.

## Data Availability

The original contributions presented in the study are included in the article/**Supplementary Material**. Further inquiries can be directed to the corresponding authors.

## Supplementary material

Supplementary Figure 1 Safety assessment after *in vivo* CB-839 treatment. **(A)** Daily body weight measurements of mice over the treatment period, n = 8-10. **(B)** Safety assessments including alanine aminotransferase (ALT), aspartate aminotransferase (AST), creatinine (CREA), lactate dehydrogenase (LDH), creatine kinase (CK), and prothrombin time (PT) levels across treatment groups,.The values are presented as the means ± standard deviations.

Supplementary Figure 2 Data-driven stratification, confounder-adjusted, and clinical sensitivity analyses. **(A)** Ranked GSE213001 IPF donor NUFIP1 expression colored by the BIC-selected Gaussian mixture classification. The smaller component contains two donors and is shown as an exploratory high-expression tail, not a validated cutoff. **(B)** BIC values for one- to three-component equal-variance **(E)** and variable-variance (V) Gaussian mixtures. Eighteen of 20 leave-one-donor-out fits selected two components. **(C)** Associations between NUFIP1 and pathway scores in a parsimonious age- and sex-adjusted HC3 model and an exploratory HC1 model additionally adjusted for smoking and disease severity. Points are beta estimates per SD higher NUFIP1; bars are robust 95% confidence intervals. **(D)** Associations of NUFIP1 with available baseline FVC (n = 19) and DLCO (n = 16). Spearman rho and BH-FDR are shown. Survival and longitudinal FVC decline were unavailable.
