# Supplementary material for: NUFIP1 gain of function promotes fibrotic remodeling and reveals a ZNHIT3-linked metabolic vulnerability in idiopathic pulmonary fibrosis models
Source: Front Immunol. 2026 Aug 27;17:1909302. doi: 10.3389/fimmu.2026.1909302 (PMC13558104; doi:10.3389/fimmu.2026.1909302)

Supplementary Source Data 1

Figure 2A

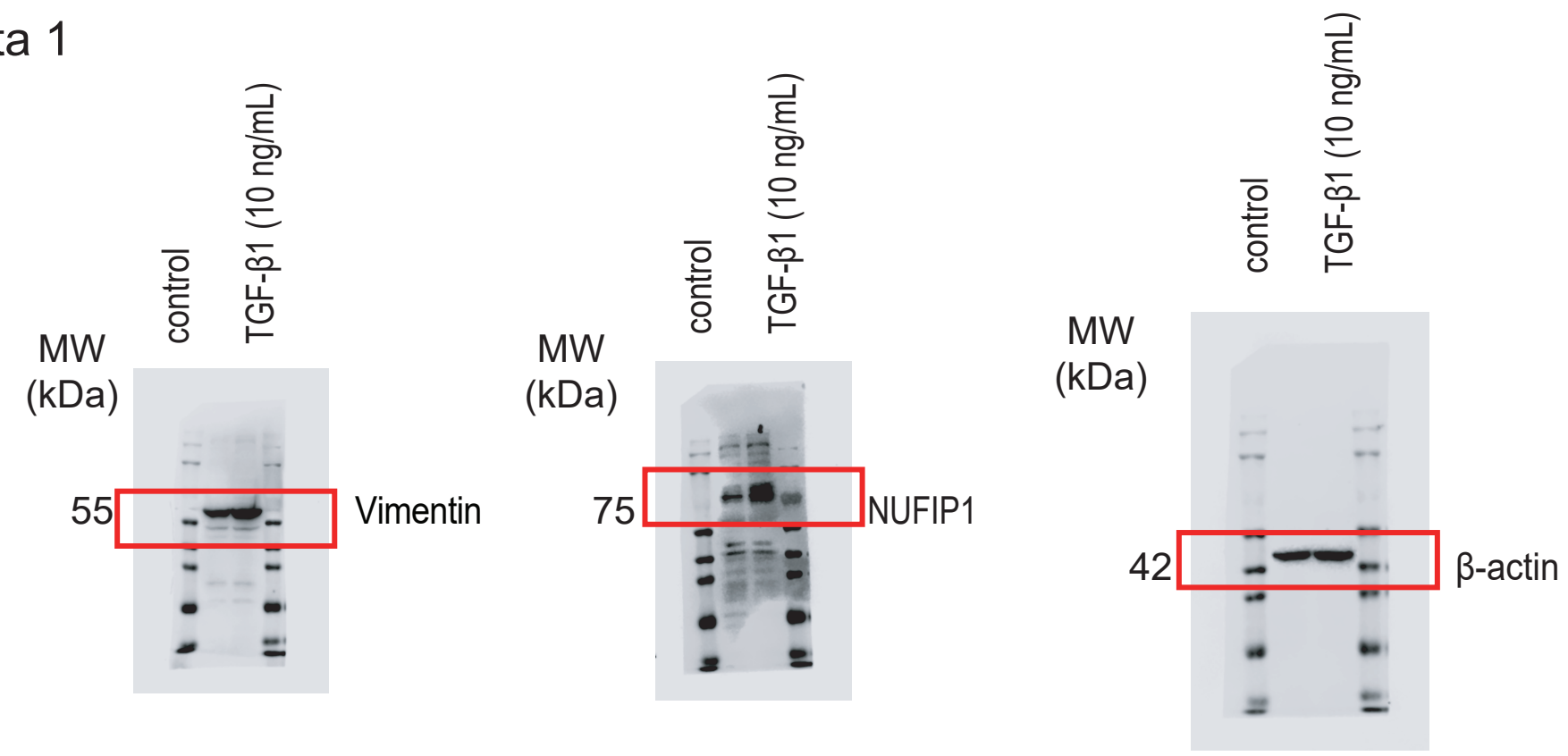

Figure 2B

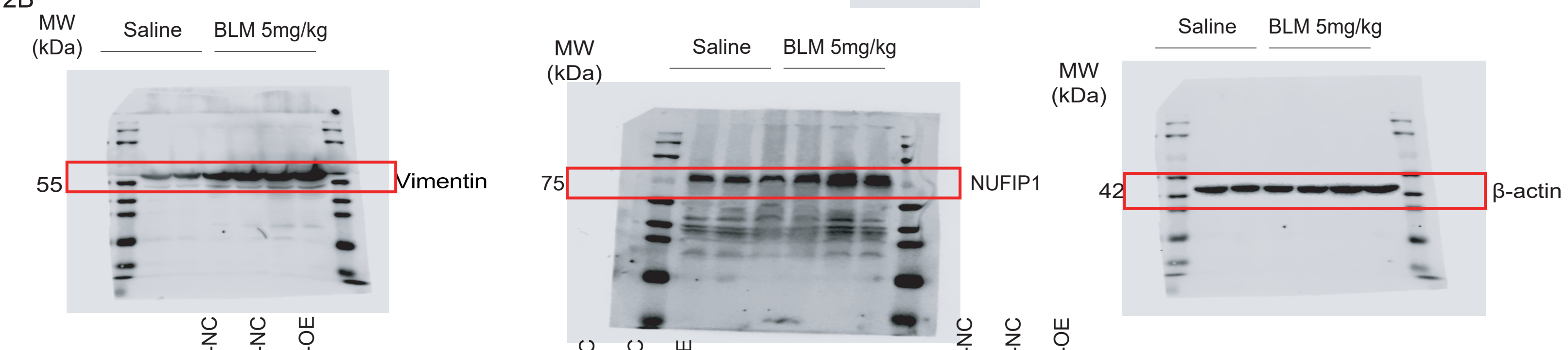

Figure 3A

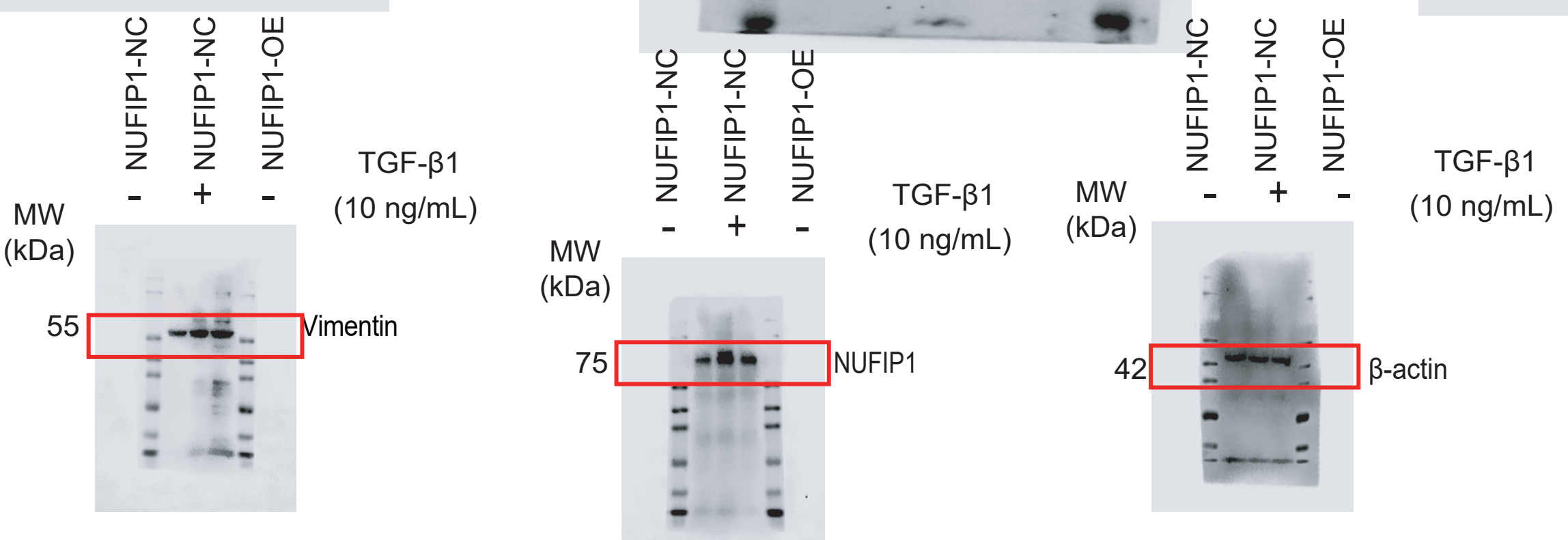

Figure 3F

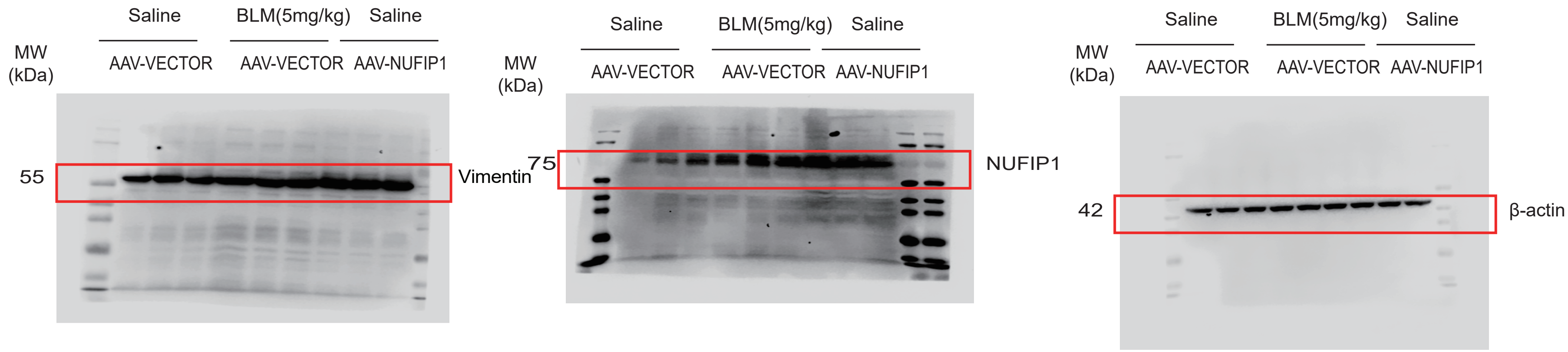

Figure 4F

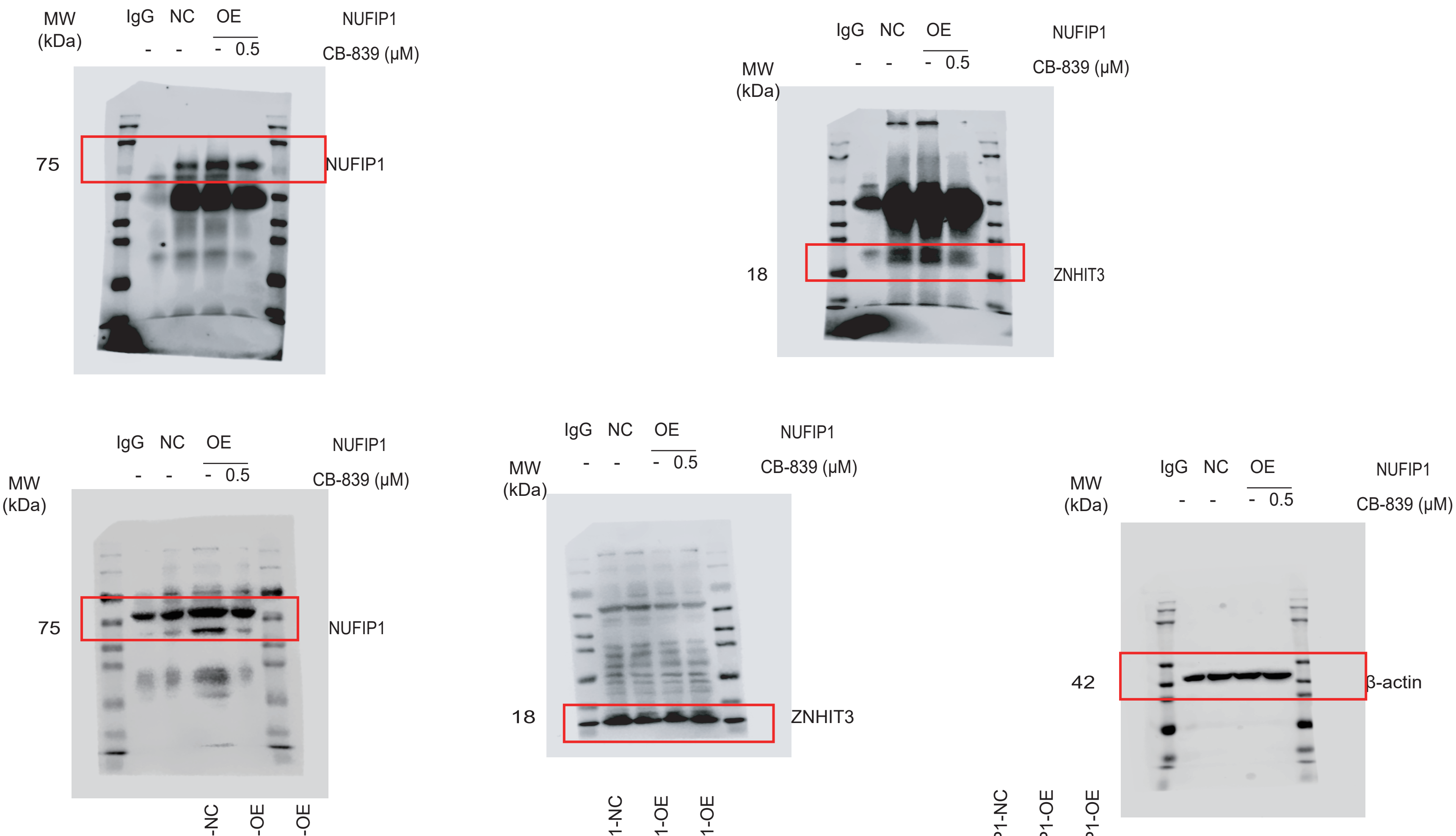

Figure 5B

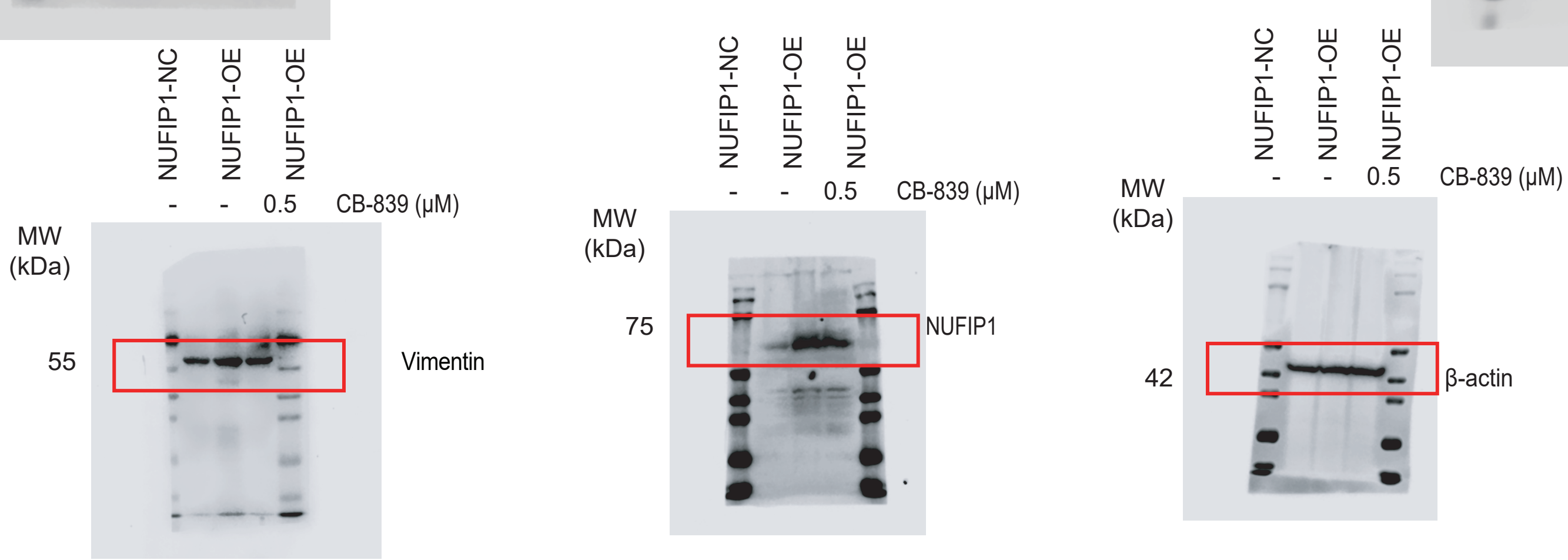

Figure 5C

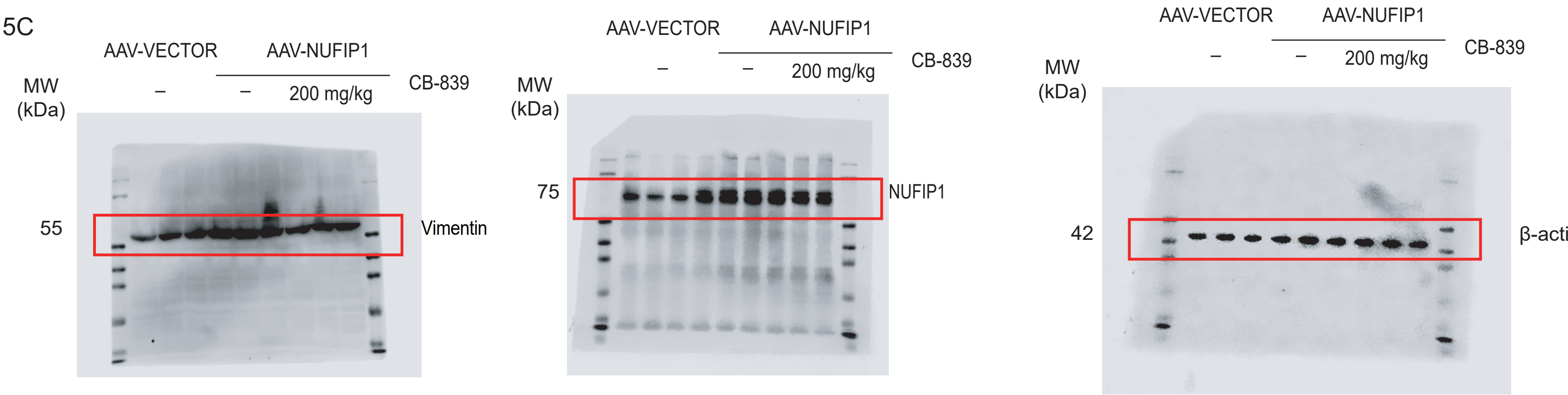

Supplement: Supplementary file 1 [file SupplementaryFile1.pdf]
