# Supplementary material for: NUFIP1 gain of function promotes fibrotic remodeling and reveals a ZNHIT3-linked metabolic vulnerability in idiopathic pulmonary fibrosis models
Source: Front Immunol. 2026 Aug 27;17:1909302. doi: 10.3389/fimmu.2026.1909302 (PMC13558104; doi:10.3389/fimmu.2026.1909302)
Supplement: Supplementary file 2 [file SupplementaryFile2.zip › reproducibility_package/figures/Figure6_revised.pdf]

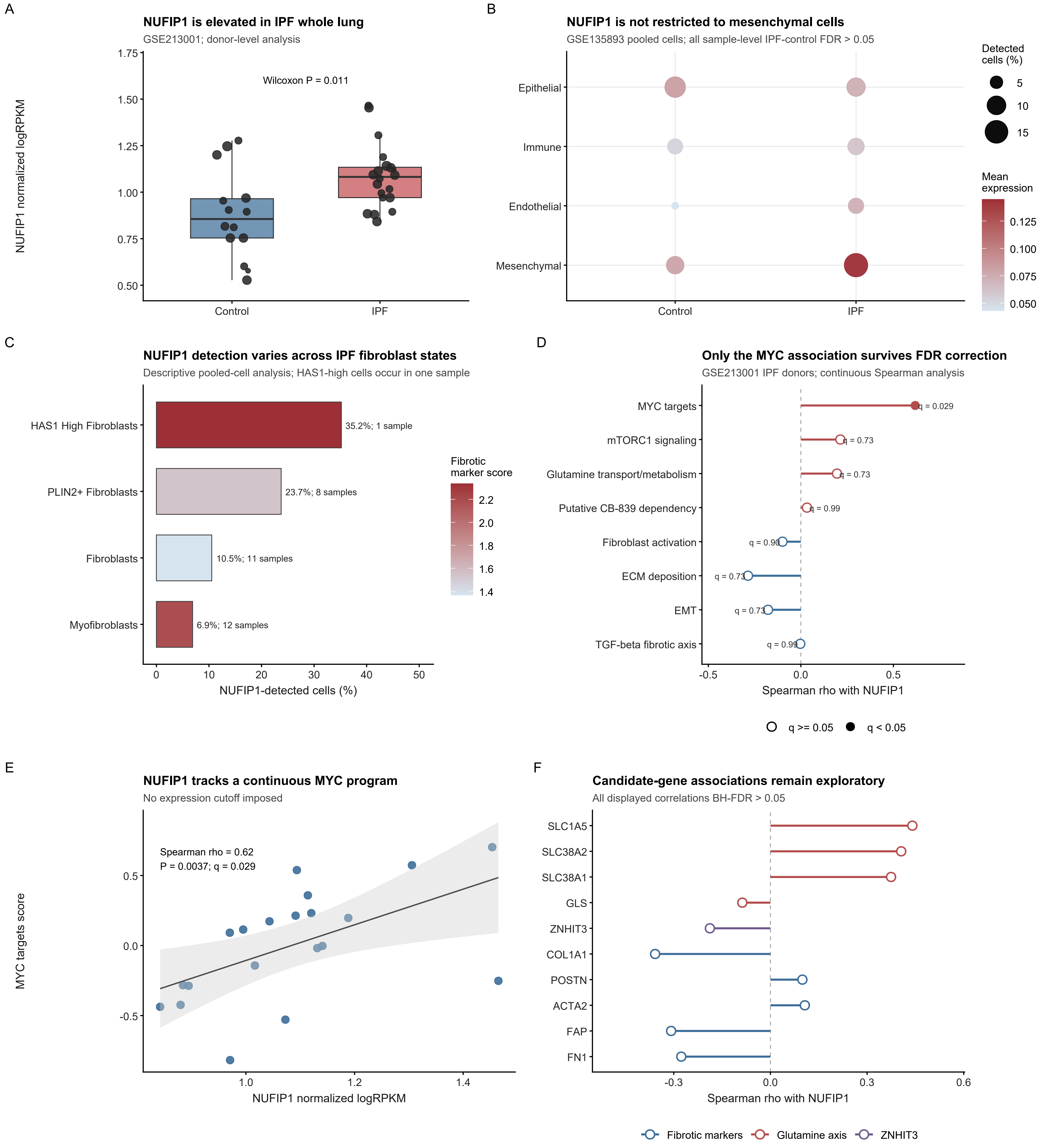

A
NUFIP1 is elevated in IPF whole lung
GSE213001; donor-level analysis
Wilcoxon P = 0.011
NUFIP1 normalized logRPKM
Control
IPF
B
NUFIP1 is not restricted to mesenchymal cells
GSE135893 pooled cells; all sample-level IPF-control FDR > 0.05
Epithelial
Immune
Endothelial
Mesenchymal
Detected cells (%)
Mean expression
C
NUFIP1 detection varies across IPF fibroblast states
Descriptive pooled-cell analysis; HAS1-high cells occur in one sample
HAS1 High Fibroblasts
PLIN2+ Fibroblasts
Fibroblasts
Myofibroblasts
35.2%; 1 sample
23.7%; 8 samples
10.5%; 11 samples
6.9%; 12 samples
Fibrotic marker score
NUFIP1-detected cells (%)
D
Only the MYC association survives FDR correction
GSE213001 IPF donors; continuous Spearman analysis
MYC targets
mTORC1 signaling
Glutamine transport/metabolism
Putative CB-839 dependency
Fibroblast activation
ECM deposition
EMT
TGF-beta fibrotic axis
q = 0.029
q = 0.73
q = 0.73
q = 0.99
q = 0.90
q = 0.73
q = 0.73
q = 0.99
Spearman rho with NUFIP1
q >= 0.05
q < 0.05
E
NUFIP1 tracks a continuous MYC program
No expression cutoff imposed
Spearman rho = 0.62
P = 0.0037; q = 0.029
MYC targets score
NUFIP1 normalized logRPKM
F
Candidate-gene associations remain exploratory
All displayed correlations BH-FDR > 0.05
SLC1A5
SLC38A2
SLC38A1
GLS
ZNHIT3
COL1A1
POSTN
ACTA2
FAP
FN1
Spearman rho with NUFIP1
Fibrotic markers
Glutamine axis
ZNHIT3
