# Supplementary material for: NUFIP1 gain of function promotes fibrotic remodeling and reveals a ZNHIT3-linked metabolic vulnerability in idiopathic pulmonary fibrosis models
Source: Front Immunol. 2026 Aug 27;17:1909302. doi: 10.3389/fimmu.2026.1909302 (PMC13558104; doi:10.3389/fimmu.2026.1909302)
Supplement: Supplementary file 2 [file SupplementaryFile2.zip › reproducibility_package/figures/FigureS2_reviewer_sensitivity.pdf]

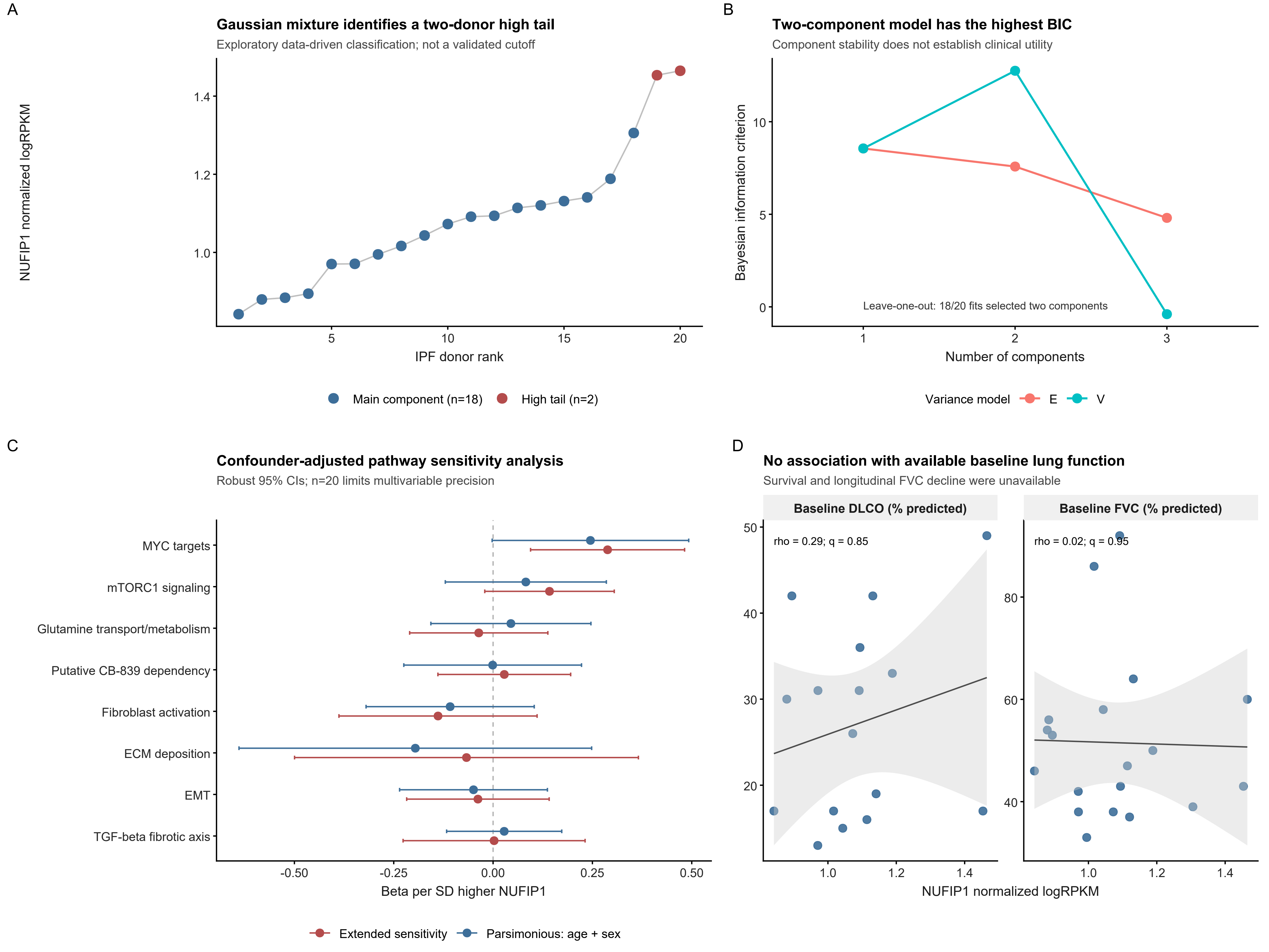
A
Gaussian mixture identifies a two-donor high tail
Exploratory data-driven classification; not a validated cutoff
NUFIP1 normalized logRPKM
1.4
1.2
1.0
5
10
15
20
IPF donor rank
Main component (n=18)
High tail (n=2)
B
Two-component model has the highest BIC
Component stability does not establish clinical utility
Bayesian information criterion
10
5
0
Leave-one-out: 18/20 fits selected two components
1
2
3
Number of components
Variance model
E
V
C
Confounder-adjusted pathway sensitivity analysis
Robust 95% CIs; n=20 limits multivariable precision
MYC targets
mTORC1 signaling
Glutamine transport/metabolism
Putative CB-839 dependency
Fibroblast activation
ECM deposition
EMT
TGF-beta fibrotic axis
-0.50
-0.25
0.00
0.25
0.50
Beta per SD higher NUFIP1
Extended sensitivity
Parsimonious: age + sex
D
No association with available baseline lung function
Survival and longitudinal FVC decline were unavailable
Baseline DLCO (% predicted)
Baseline FVC (% predicted)
rho = 0.29; q = 0.85
rho = 0.02; q = 0.95
50
40
30
20
80
60
40
1.0
1.2
1.4
NUFIP1 normalized logRPKM
